# International trends of orthodontic publications: A bibliometric observational study of the last decade (2011-2020)

**DOI:** 10.1590/2177-6709.28.1.e2321175.oar

**Published:** 2023-04-03

**Authors:** Nabeel ALMOTAIRY

**Affiliations:** 1 Qassim University, College of Dentistry, Department of Orthodontics and Pediatric Dentistry (Buraidah, Saudi Arabia).

**Keywords:** Orthodontics, Bibliometrics, Article characteristics, Orthodontic journals, Dental research

## Abstract

**Introduction:**

The number of published orthodontic studies has increased considerably over the past ten years.

**Objective:**

To analyze the bibliometric data of international orthodontic studies included in orthodontic journals encompassed by the Scopus database between 2011 and 2020, as well as to undertake data comparison between the period 2010-2015 and the period 2016-2020.

**Materials and methods:**

A retrospective search was conducted on 14 orthodontic journals included in the Scopus database from 2011 to 2020. Studies of both primary and secondary types were targeted by the search. The yearly number of studies published in the 14 journals, and the first 20 countries, institutions and their type (public/private), and authors, respectively, regarding publication volume, were presented.

**Results:**

Over the past ten years, the number of publications in the chosen journals reached 9200, where the American Journal of Orthodontics and Dentofacial Orthopedics and Angle Orthodontist topped the journal list, with 22% and 12% of the publications, respectively. Furthermore, the orthodontic publication volume showed a declining trend by the end of the decade (-9%), where academic/public institutions produced most of the orthodontic studies, and the US (20%), Brazil (17%), and South Korea (8%) topped the countries with the most orthodontic studies. A comparison of the two halves of the decade revealed that orthodontic research exhibited an increasing trend in developing nations, especially Egypt (104%), Saudi Arabia (88%), and Iran (83%).

**Conclusion:**

The orthodontic studies published in the chosen journals over the past ten years showed a dynamic change in yearly publication and ranking of countries, institutions, and authors.

## RESUMO

**Introdução:**

O número de estudos publicados na área da Ortodontia aumentou consideravelmente nos últimos dez anos.

**Objetivos:**

Analisar os dados bibliométricos dos estudos ortodônticos internacionais publicados entre 2011 e 2020 em periódicos ortodônticos da base de dados Scopus, bem como realizar a comparação entre os dados dos períodos 2010-2015 e 2016-2020.

**Material e Métodos:**

Uma pesquisa retrospectiva foi realizada em 14 periódicos ortodônticos incluídos na base de dados Scopus de 2011 a 2020. Estudos dos tipos primários e secundários foram incluídos na pesquisa. Foram apresentados o número anual de estudos publicados em 14 revistas e os 20 primeiros países, instituições e seu tipo (público/privado) e autores, respectivamente, em relação ao volume de publicações.

**Resultados:**

Nos últimos dez anos, o número de publicações nos periódicos escolhidos chegou a 9.200, e o *American Journal of Orthodontics and Dentofacial Orthopedics* e o *Angle Orthodontist* lideraram a lista de periódicos, com 22% e 12% das publicações, respectivamente. Além disso, o volume de publicações ortodônticas apresentou uma tendência de queda no final da década (-9%), sendo que as instituições acadêmicas/públicas produziram a maior parte dos estudos ortodônticos, e os EUA (20%), Brasil (17%) e a Coreia do Sul (8%) lideraram os países com mais estudos ortodônticos. Uma comparação das duas metades da década revelou que a pesquisa ortodôntica exibiu uma tendência crescente nos países em desenvolvimento, especialmente Egito (104%), Arábia Saudita (88%) e Irã (83%).

**Conclusão:**

Os estudos ortodônticos publicados nos últimos dez anos nos periódicos escolhidos mostraram uma mudança dinâmica na publicação anual e no ranking de países, instituições e autores.

## INTRODUCTION

Scholarly publication undergoes continuous change with time, and has increased over the recent decades.^1^ The continuous change in the bibliometric data of such publications (such as author characteristics, publication type, affiliation, and country of origin) can be described with bibliometric methods. Such methods can shed light on the effects elicited by a particular domain, group of authors, and subjects, and identify studies of special significance in a particular research field.

There has been a marked rise in the number of orthodontic publications in recent times due to innovations in clinical procedures, applications, and techniques.^2^ ^,^ ^3^ The bibliometric data of orthodontic studies have been the focus of a few bibliometric studies, with emphasis on distinct research questions, time-frame periods, databases, and journal breadth.^3^ ^-^ ^20^ For instance, the study by Tarazona-Alvarez et al^4^ was concerned solely with the bibliometric data of lingual orthodontics studies, whereas other studies analyzed the bibliometric data of orthodontic studies conducted in particular parts of the world^15^ ^,^ ^18^ or that adopted specific research designs, such as systematic reviews and/or randomized clinical trials.^3^ ^,^ ^17^ Moreover, highly cited orthodontic studies were explored within particular intervals of recent decades.^7^ ^,^ ^9^ ^,^ ^20^ Certain bibliometric studies concentrated on publications from a limited number of orthodontic journals^9^ ^,^ ^13^ ^,^ ^18^ ^,^ ^19^ or database range.^3^ ^,^ ^6^ ^,^ ^14^ ^,^ ^18^ However, the restrictions in database coverage or in the number of included orthodontic journals may affect the interpretation of orthodontic literature bibliometrics, thus limiting the studies’ outcome generalizability. Therefore, the present study intended to include as many orthodontic journals as possible in order to gain as broad a picture of orthodontic literature as possible.

The majority of previous orthodontic bibliometric studies have used Web of Science database (WoS).^4^ ^,^ ^5^ ^,^ ^7^ ^-^ ^9^ ^,^ ^16^ ^,^ ^19^ ^,^ ^20^ As far as the author is aware, the bibliometrics of orthodontic studies published in the journals encompassed in Scopus database have never been investigated. Indeed, the Scopus database does not have the longevity of the WoS, having been created in 2004, but displays greater breadth.^21^ Regarding orthodontic literature, WoS encompasses 13 orthodontic journals as of 2021, of which 50% were added since 2015. On the other hand, 17 orthodontic journals are contained in Scopus, and most were added before 2005. Thus, the Scopus database compared to the WoS is an optimum choice when conducting orthodontic bibliometric studies due to its inclusion of more orthodontic journals with a greater literature coverage.

Given the above considerations, the purpose of the present paper was to explore bibliometric data of international orthodontic studies publications in orthodontic journals included in the Scopus database in the period from 2011 to 2020, as well as to compare how the bibliometric data transformed dynamically between the two halves of the decade in question.

## MATERIAL AND METHODS

### LITERATURE SEARCH STRATEGY

The paper adopted an observational research design to review international orthodontic studies published in the last ten years based on the Scopus publication bibliometrics. The search was conducted on April 1st, 2021, by searching for orthodontic journals included in Scopus for the period between 2011 and 2020. The chosen journals were the American Journal of Orthodontics and Dentofacial Orthopedics (AJODO), Angle Orthodontist (AO), Australasian Orthodontic Journal (AOJ), Dental Press Journal of Orthodontics (DPJO), European Journal of Orthodontics (EJO), International Orthodontics (IO), Journal of Clinical Orthodontics (JCO), Journal of Orofacial Orthopedics (JOO), Journal of Orthodontics (JO), Journal of the World Federation of Orthodontists (JWFO), Korean Journal of Orthodontics (KJO), Orthodontic Waves (OW), Orthodontics & Craniofacial Research (OCR), and Progress in Orthodontics (PO). The chosen journals were subjected to a retrospective search for studies published in the last ten years based on the Scopus advanced search tool. This method involved determining the database name indices of the target orthodontic journals and subsequently merging those indices with Boolean operators, as follows.

( SRCTITLE ( American AND Journal AND of AND Orthodontics AND Dentofacial AND Orthopedics ) ) OR ( SRCTITLE ( European AND Journal AND of AND Orthodontics ) ) OR ( SRCTITLE ( Angle AND Orthodontist ) ) OR ( SRCTITLE ( Progress AND in AND Orthodontics ) ) OR ( SRCTITLE ( Korean AND Journal AND of AND Orthodontics ) ) OR ( SRCTITLE ( Journal AND of AND Orofacial AND Orthopedics ) ) OR ( SRCTITLE ( Orthodontics AND Craniofacial AND Research ) ) OR ( SRCTITLE ( Dental AND Press AND Journal AND of AND Orthodontics ) ) OR ( SRCTITLE (Journal AND of AND Clinical AND Orthodontics)) OR ( SRCTITLE ( International AND Orthodontics ) ) OR ( SRCTITLE ( Journal AND of AND Orthodontics ) ) OR ( SRCTITLE ( Journal AND of AND The AND World AND Federation AND of AND Orthodontists ) ) OR ( SRCTITLE ( Orthodontic AND Waves ) ) OR ( SRCTITLE ( Australasian AND Orthodontic AND Journal ) ) OR ( SRCTITLE ( Australian AND Orthodontic AND Journal ) ) AND ( LIMIT-TO ( DOCTYPE, “ar”) OR LIMIT-TO ( DOCTYPE, “re”) ) AND ( LIMIT-TO ( PUBYEAR,2020) OR LIMITTO ( PUBYEAR,2019) OR LIMIT-TO ( PUBYEAR,2018) OR LIMIT-TO ( PUBYEAR,2017) OR LIMIT-TO ( PUBYEAR,2016) OR LIMIT-TO ( PUBYEAR,2015) OR LIMIT-TO ( PUBYEAR,2014) OR LIMIT-TO ( PUBYEAR,2013) OR LIMIT-TO ( PUBYEAR,2012) OR LIMIT-TO ( PUBYEAR,2011) ) AND ( EXCLUDE ( EXACTSRCTITLE, “Journal of Orthodontic Science”) ) AND ( EXCLUDE ( EXACTSRCTITLE, “International Journal of Orthodontics Milwaukee Wis”) OR EXCLUDE ( EXACTSRCTITLE, “Turkish Journal of Orthodontics”) )

Orthodontic journals were excluded if they lacked complete or partial Scopus database coverage for the period in question. Such journals included the Turkish Journal of Orthodontics, Journal of Orthodontic Sciences and Journal of Orthodontics Milwaukee Wisconsin. Note that no language restrictions were put during the database search.

### ELIGIBILITY CRITERIA

Original and review studies published between 2011 and 2020 were included, but biographies, book reviews, book chapters, editorials, retractions, errata, and proceedings were excluded. 

### DATA EXTRACTION

The bibliometric data associated with the orthodontic studies were exported using the Scopus database’s refine result’ tool. This tool was used to extract the number of studies published every year during 2011-2020 and the number of studies published in every chosen journal, country, institution, and authors. The tool was also used to extract the proportion of narrative versus systematic reviews and the publication language of the retrieved studies. Furthermore, the ‘view citation overview’ tool of the Scopus database was employed to extract the citation data of all authors, including the rate of self-citation during 2011-2020. 

The search result was exported to Microsoft Excel, and descriptive statistics were produced regarding the yearly number of studies published in the 14 journals and first 20 countries, institutions and their type (public/private), and authors in terms of publication volume. Additionally, data comparison of the selected variables was also conducted between the two halves of the decade (2011-2015 versus 2016-2020).

## RESULTS

The number of orthodontic publications included in the chosen journals in the last ten years was 9200. Of these, 8802 were articles and 398 were reviews. The reviews comprised two sub-types: systematic reviews (n = 327) and narrative reviews (n = 71). The language of the greatest proportion of articles (94%) was English, whilst 4% of articles were written in French and 1.5% in German. By contrast, articles written in Korean and Portuguese accounted for less than 0.5% of the overall publications. The source journal, year, country, institution, and author were the criteria used to organize the descriptive data pertaining to the orthodontic studies.

## PUBLICATION JOURNAL

The number of publications in the chosen orthodontic journals over the past ten years is given in Table 1. AJODO contained 22% of the identified studies, while AO contained 14%. Compared to the first half of 2011-2020, fewer studies were published in the AJODO, AO, EJO, DPJO, and AOJ in the second half. 

**Table 1:** The number and proportion of publications in the chosen orthodontic journals over the last ten years and during the first and second parts of the decade.

Rank	Journal	Total	%	2011-2015		2016-2020		Trend change (%)
				Articles	%	Articles	%	
1	American Journal of Orthodontics and Dentofacial Orthopedics	2009	22	1102	23	907	20	-18
2	Angle Orthodontist	1311	14	741	16	570	13	-23
3	European Journal of Orthodontics	952	10	539	11	413	9	-23
4	Dental Press Journal of Orthodontics	848	9	540	11	308	7	-43
5	Journal of Clinical Orthodontics	717	8	355	7	362	8	2
6	International Orthodontics	587	6	203	4	384	9	89
7	Orthodontics & Craniofacial Research	433	5	156	3	277	6	78
8	Progress in Orthodontics	421	5	202	4	219	5	8
9	Journal of Orthodontics	420	5	178	4	242	5	36
10	Korean Journal of Orthodontics	408	4	197	4	211	5	7
11	Journal of Orofacial Orthopedics	396	4	194	4	202	5	4
12	Australasian Orthodontic Journal	254	3	132	3	122	3	-8
13	Journal of The World Federation of Orthodontists	236	3	104	2	132	3	27
14	Orthodontic Waves	208	2	95	2	113	3	19

However, other journals had either the same or a higher research output in the second half of the decade by comparison to the first.

## PUBLICATION YEAR

**Figure 1:**
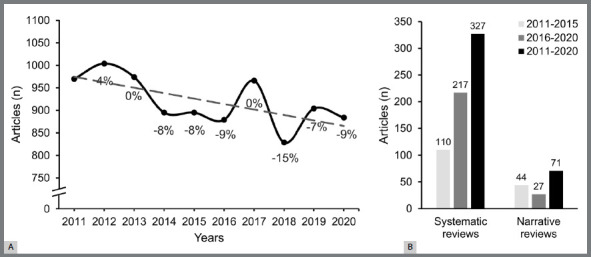
A) The number of publications between 2011 and 2020 alongside the relative change in the proportion of publications compared to 2011; B) The overall number of narrative and systematic reviews published between 2011 and 2020 as well as during the first and second parts of the decade.

**Figure 2:**
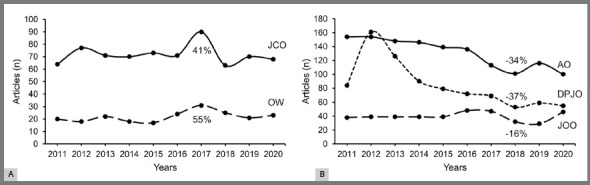
A) The number of publications between 2011 and 2020 by JCO and OW journals alongside their relative change in the proportion of publications in 2017 compared to 2011; B) The number of publications between 2011 and 2020 by DPJO, AO and JOO journals alongside their relative change in the proportion of publications in 2018 compared to 2011.

Figure 1A shows the yearly volume of orthodontic publications during 2011-2020, where the number of publications was 9% lower in 2020 than in 2011. In 2017, the number of orthodontic publications was the same as in 2011, mainly due to the increased research output of JCO and OW (Fig 2A). While the number of orthodontic publications in 2018 relative to 2011 decreased by 15%, mainly due to the reduced research output of DPJO, AO, and JOO (Fig 2B). The number of published reviews in 2011-2015 and 2016-2020 is presented in Figure 1B. In the first half of the decade, the proportion of reviews accounted for 3% of the overall number of studies, whereas in the second half, it accounted for 5.5%. However, even though systematic reviews published in 2016-2020 were twice as many as those published in 2011-2015, the number of narrative reviews declined by 38% for the same interval.

## PUBLICATION COUNTRY

The orthodontic studies published in the last ten years were produced in 107 countries, especially the US (20%), Brazil (17%), South Korea (8%), Italy (7%), and the UK (7%). The first 20 countries with the greatest proportion of publications in the chosen journals are listed in Table 2. Comparison of the two halves of the decade was conducted to assess changes in research output trend in the foremost 20 countries. Thus, a number of eight countries displayed a downturn in research output, yet most countries displayed a rise in research output, particularly Egypt, with an increase of 104%, Saudi Arabia (88%), Iran (83%), and Australia (63%).

**Table 2:** The first 20 countries in terms of orthodontic research output, alongside alterations in their ranking and publication yield in 2011-2015 and 2016-2020.

Rank	Country	Total	2011-2015		2016-2020		Trend change (%)
			Rank	Articles	Rank	Articles	
1	United States	1869	1	951	1	918	-3
2	Brazil	1552	2	897	2	655	-27
3	South Korea	705	3	363	3	342	-6
4	Italy	624	4	316	4	308	-3
5	United Kingdom	604	6	297	5	307	3
6	Germany	525	5	305	9	220	-28
7	Japan	522	7	285	6	237	-17
8	Turkey	486	8	257	7	229	-11
9	China	386	10	164	8	222	35
10	India	365	9	180	10	185	3
11	Switzerland	323	11	139	11	184	32
12	Canada	304	12	132	12	172	30
13	France	200	13	102	18	98	-4
14	Greece	198	14	88	15	110	25
15	Australia	191	16	73	13	118	62
16	Iran	178	18	63	14	115	83
17	Egypt	161	20	53	16	108	104
18	Saudi Arabia	161	19	56	17	105	88
19	Sweden	154	15	74	19	80	8
20	Netherlands	137	17	66	20	71	8

## PUBLICATION INSTITUTION

Over the last ten years, orthodontic research was carried out across 8022 institutions. The first 20 institutions with the greatest proportion of publications in the chosen journals, alongside research output changes and rank during 2011-2015 and 2016-2020 are listed in Table 3. The foremost five institutions were: Universidade de Sao Paulo in Brazil (n = 444 studies), Universidade Federal do Rio de Janeiro (n = 208 studies), Kyung Hee University (n = 190 studies), University of Alberta (n = 183 studies), and Seoul National University (n = 159 studies).

**Table 3:** The first 20 institutions in terms of orthodontic research output, alongside alterations in their ranking and publication yield in 2011-2015 and 2016-2020.

Rank	Affiliation	Articles	2011-2015		2016-2020	
			Rank	Articles	Rank	Articles
1	Universidade de São Paulo (USP)	444	1	227	1	217
2	Universidade Federal do Rio de Janeiro	208	2	140	7	68
3	Kyung Hee University	190	5	87	2	103
4	University of Alberta	183	7	80	3	103
5	Seoul National University	159	4	91	8	68
6	Universidade Estadual Paulista (UNESP)	156	3	94	10	62
7	University of Michigan, Ann Arbor	148	9	75	6	73
8	University of Zurich	147	13	57	4	90
9	University of Bern	147	6	80	9	67
10	Yonsei University	139	10	65	5	74
11	Università degli Studi di Firenze	120	12	63	12	57
12	National and Kapodistrian University of Athens	113	15	56	13	57
13	Universität Bonn	104	14	56	20	48
14	Universidade do Estado do Rio de Janeiro	103	8	75	45	28
15	St. Louis University	103	19	49	14	54
16	The University of North Carolina at Chapel Hill	102	17	49	15	53
17	University of Ferrara	100	16	53	22	47
18	Arizona School of Dentistry and Oral Health	98	28	37	11	61
19	Texas A&M University System	96	11	64	39	32
20	The Catholic University of Korea	89	17	50	29	39

## PUBLICATION AUTHOR

The number of authors involved in the production of the publications in the chosen orthodontic journals over the last ten years was 11,957. Co-authorship with at least one author was the norm in most of the studies. The first 20 authors with the greatest proportion of publications in the chosen orthodontic journals are provided in Table 4. The maximum number of studies (n = 144) was authored by Guilherme Janson, while the next four authors with the highest number of studies were Lorenzo Franchi (n = 102 studies), Carlos Flores-Mir (n = 99 studies), Nikolaos Pandis (n = 93 studies), and Jae Hyun Park (n = 93 studies). 

**Table 4:** The first 20 authors with the greatest proportion of publications in the chosen journals, as well as the proportion of citation, including self-citation, over the last ten years.

Rank	Author	Articles	Citations												Self-cite (%)
			2011	2012	2013	2014	2015	2016	2017	2018	2019	2020	2021	Total	
1	Janson, G	144	0	10	20	37	76	80	101	129	148	213	107	921	21
2	Franchi, L	102	8	23	45	60	89	135	173	226	254	323	145	1481	22
3	Flores-Mir, C	99	0	4	12	40	49	96	128	148	188	257	112	1034	13
4	Pandis, N	93	0	8	33	79	106	120	126	145	216	264	111	1208	21
5	Park, JH	93	2	4	17	18	53	48	79	100	98	161	95	675	33
6	Eliades, T	92	1	8	32	65	83	86	103	132	181	228	114	1033	23
7	Nanda, R	86	1	6	6	30	53	66	68	118	121	118	59	646	14
8	Pithon, MM	86	0	4	11	35	39	69	68	59	104	89	33	511	18
9	Buschang, PH	77	5	18	54	85	107	100	148	138	155	207	101	1118	10
10	Lombardo, L	72	1	3	10	10	13	43	63	83	77	102	57	462	17
11	Siciliani, G	68	0	2	9	17	20	45	72	80	84	111	58	498	14
12	Baek, SH	67	4	12	27	43	60	46	56	79	53	84	45	509	18
13	Kim, SH	67	6	15	39	81	57	60	78	77	92	90	75	670	27
14	Kook, YA	67	4	10	29	36	96	71	101	135	119	163	107	871	35
15	Papageorgiou SN	64	0	2	18	33	59	65	101	138	175	231	109	931	22
16	Bourauel, C	61	2	4	16	33	50	67	91	84	92	104	58	601	18
17	Consolaro, A	57	0	1	3	2	7	19	23	26	46	55	18	200	13
18	Darendeliler, MA	54	4	10	22	27	55	59	59	71	97	88	46	538	12
19	Jäger, A	54	2	8	12	31	32	53	73	84	72	90	52	509	24
20	Katsaros, C	53	1	11	38	70	94	97	119	119	114	144	52	859	17

The overall rate of self-citation was 19.6%, where 50% of the first 20 authors cited themselves in proportion of 10-20% whilst the other 50% cited themselves in proportion of 21-35%.

## DISCUSSION

Orthodontic studies proliferated in the past years. Bibliometric studies could be employed to observe how trends in publication changed dynamically. The bibliometric data associated with international orthodontic publications in orthodontic journals included in the Scopus database over the period between 2011 and 2020 were analyzed in the present paper. In addition, a comparison was conducted between the two halves of the decade in question about the transformations in bibliometric data. As far as the author is aware, this is the first paper to comprehensively explore the orthodontic studies published in journals encompassed in the Scopus database over the last ten years.

The bibliometrics of published orthodontic studies were analyzed in a number of Studies.^3^ ^-^ ^20^ Of those studies, some focused on citation patterns or investigated subjects,^3^ ^,^ ^7^ ^,^ ^9^ ^,^ ^12^ ^,^ ^17^ ^,^ ^20^ while other studies focused on the bibliometric data of orthodontic studies published only in limited orthodontic journals.^9^ ^,^ ^13^ ^,^ ^18^ ^,^ ^19^ Thus, the current study chose 14 popular orthodontic journals encompassed in the Scopus database in the period between 2011 and 2020 to gain as broad a picture of orthodontic publication trends as possible. Furthermore, distortion of results was prevented by excluding journals that were included only partially in the Scopus database.

One major observation derived from this analysis was that the number of reviews rose from 2011-2015 to 2016-2020 primarily as a result of the increase in systematic reviews in the second half of the decade. In recent times, systematic reviews have increased, where they were published more often than clinical trials due to several reasons.^22^ On average, systematic reviews require less resources, can take a shorter time to finish, and bring more citations than clinical trials.^22^ Systematic reviews also require no ethical clearance, which makes them convenient for postgraduate research projects.^22^ Regardless of the increase in systematic reviews, there was a decrease of about 9% in the total number of orthodontic studies by the end of the decade. Five journals were particularly associated with this decrease, namely, AJODO, AO, EJO, DPJO, and AOJ, whereas the research output of the other nine journals remained unchanged or rose to a minor extent. It must be highlighted that the findings obtained are applicable solely to the chosen journals, they do not reflect a reduction in the general proportion of orthodontic studies. As indicated by Mavropoulos and Kiliaridis, studies pertaining to the topic of orthodontics can be published in journals that are not exclusively dedicated to the topic.^6^ Indeed, the authors showed that such non-specialist journals received a fair number of orthodontic studies. This could be one reason for the decrease in orthodontic research output discovered in the present paper, as can the proliferation of online open access journals with wide coverage, which would have captured a greater proportion of orthodontic studies. Moreover, the Covid-19 pandemic coupled with the introduced safety measures could have contributed to the decline in orthodontic publications in 2020 by restricting the possibility of undertaking clinical research. However, the pandemic must be fully resolved before an assessment can be made of its implications for the orthodontic research output.

As far as the publication country is concerned, this paper corroborated earlier investigations by finding that most published studies were produced in the US.^15^ ^,^ ^16^ ^,^ ^19^ followed by Brazil, South Korea, Italy, and the UK. However, the paper differs from earlier investigations in the fact that discrepancies in chosen journals and time periods engendered variability in the number of studies and the ranking of the publication countries.^15^ ^,^ ^16^ ^,^ ^19^ Furthermore, in partial agreement with earlier work,^8^ ^,^ ^15^ ^,^ ^16^ comparison between the two halves of the decade in question revealed that developing countries (e.g. China, Iran, Egypt, Saudi Arabia) exhibited an increasing trend in the number of orthodontic studies. This increase could be attributed to convoluted personal, institutional, and national determinants, such as modifications in governmental policies, greater research funds, improvement in research infrastructure, the financial potential of academia, and enhanced rate of postgraduate registration.^23^

Another point of consistency between this paper and earlier work is the finding that academic institutions produced most orthodontic studies.^8^ ^,^ ^16^ ^,^ ^17^ ^,^ ^19^ ^,^ ^24^ According to Aura et al.,^16^ academic institutions generated 80% of the orthodontic studies published in the journals included in the Journal Citation Report from 2007 to 2017, while non-academic and private institutions showed a decreasing trend in orthodontic research output.^16^ One explanation for such results could be the greater emphasis placed on intensification of orthodontic research programs and recruitment of well-qualified academics with ample research experience to attain the necessary funding.^6^ The favorable effects of specialty program accreditations like the Network of Erasmus‐Based European Orthodontic Postgraduate Programs could also elucidate the high number of orthodontic studies produced by academic institutions.^25^

The current results also showed that the overall self-citation rate was 19.6%, corroborating what was previously reported in orthodontics,^16^ ^,^ ^26^ dental,^27^ and other medical fields.^28^ According to Livas et al.^26^, author origin and gender were strong contributors to the self-citation rate, where Asian authors and females were less likely to cite themselves than other regions and male authors. In the present study, the self-citation rate of the first 20 authors was registered (Table 4), but no intention was put to profoundly investigate the self-citation pattern since most authors publish studies in other journals/fields, which could lead to inaccurate interpretation and comparison with other studies.

The present paper is not without shortcomings, such as the limitation of the number of orthodontic journals to just 14. Journals covering a wide variety of disciplines have started to proliferate,^29^ with 287 such journals have been founded by the MDPI publisher, as an example. Over the past ten years, MDPI publications have increased annually, with around 110,000 articles being published just in 2019.^12^ Hence, it is highly likely that studies on the topic of orthodontics could have been included in those journals. At the same time, it could be challenging to differentiate articles related to specialties from articles without any particular specialty. The journals examined in this paper were chosen specifically to capture as many orthodontic studies as possible while screening out non- specialty studies. Nevertheless, open access journals with wide coverage should be included in future research in order to better understand the pattern of published orthodontic studies.

## CONCLUSION

The number of orthodontic studies in the chosen journals decreased, whereas the number of systematic reviews increased over the last ten years. The AJODO, AO, EJO, and DPJO accounted for over 50% of the number of orthodontic publications over the period in question. Academic and public institutions produced the largest proportion of studies, and the top five countries in which orthodontic studies were conducted were the US, Brazil, South Korea, Italy, and the UK. A comparison of the two halves of the decade in question showed that the number of orthodontic studies was on the rise in developing countries, including China, Iran, Egypt, and Saudi Arabia.
